# On the Origin of Muscle Synergies: Invariant Balance in the Co-activation of Agonist and Antagonist Muscle Pairs

**DOI:** 10.3389/fbioe.2015.00192

**Published:** 2015-11-24

**Authors:** Hiroaki Hirai, Fumio Miyazaki, Hiroaki Naritomi, Keitaro Koba, Takanori Oku, Kanna Uno, Mitsunori Uemura, Tomoki Nishi, Masayuki Kageyama, Hermano Igo Krebs

**Affiliations:** ^1^Department of Mechanical Science and Bioengineering, Graduate School of Engineering Science, Osaka University, Toyonaka, Japan; ^2^Department of Neurology, Senri Chuo Hospital, Toyonaka, Japan; ^3^Department of Rehabilitation, Senri Chuo Hospital, Toyonaka, Japan; ^4^Department of Mechanical Engineering, Massachusetts Institute of Technology, Cambridge, MA, USA; ^5^Department of Neurology, University of Maryland School of Medicine, Baltimore, MD, USA; ^6^Department of Rehabilitation Medicine I, School of Medicine, Fujita Health University, Toyoake, Japan; ^7^Institute of Neuroscience, University of Newcastle, Newcastle upon Tyne, UK

**Keywords:** muscle synergy, motor primitives, mechanical impedance, reference frame, virtual trajectory, endpoint stiffness, electromyography

## Abstract

Investigation of neural representation of movement planning has attracted the attention of neuroscientists, as it may reveal the sensorimotor transformation essential to motor control. The analysis of muscle synergies based on the activity of agonist–antagonist (AA) muscle pairs may provide insight into such transformations, especially for a reference frame in the muscle space. In this study, we examined the AA concept using the following explanatory variables: the AA ratio, which is related to the equilibrium-joint angle, and the AA sum, which is associated with joint stiffness. We formulated muscle synergies as a function of AA sums, positing that muscle synergies are composite units of mechanical impedance. The AA concept can be regarded as another form of the equilibrium-point (EP) hypothesis, and it can be extended to the concept of EP-based synergies. We introduce, here, a novel tool for analyzing the neurological and motor functions underlying human movements and review some initial insights from our results about the relationships between muscle synergies, endpoint stiffness, and virtual trajectories (time series of EP). Our results suggest that (1) muscle synergies reflect an invariant balance in the co-activation of AA muscle pairs; (2) each synergy represents the basis for the radial, tangential, and null movements of the virtual trajectory in the polar coordinates centered on the specific joint at the base of the body; and (3) the alteration of muscle synergies (for example, due to spasticity or rigidity following neurological injury) results in significant distortion of endpoint stiffness and concomitant virtual trajectories. These results indicate that muscle synergies (i.e., the balance of muscle mechanical impedance) are essential for motor control.

## Introduction

Voluntary movement requires sensorimotor transformation between extrinsic and intrinsic frames of reference (Kandel et al., 2012). To execute movement with specific endpoint characteristics, including aspects of kinematics, force, and impedance, the sensorimotor transformation may directly map the muscle space into the task space; the central nervous system (CNS) needs to regulate muscle activities to meet the endpoint’s kinematic and kinetic specification. If the muscle space directly relates to the task space, the endpoint movement could be planned or predicted based on the reference frame in the muscle space in which the motor commands from the CNS to the muscles are encoded. The reference frame in the muscle space provides a framework to explain how humans plan, adjust, and achieve a desired endpoint movement when governing multiple muscles in the executing limb.

However, the neuromusculoskeletal system is neurologically and mechanically redundant. The inverse problem (i.e., movement planning) involves an infinite number of possible solutions to a given task. One hypothesis for solving this ill-posed problem is to exploit the stereotypical patterns of coordination, or muscle synergies. Synergies are classes of movement patterns that are functional groups of structural elements in the regulation and control of movement (Bernstein, 1967). The synergy hypothesis emphasizes that the CNS utilizes the functional structure at different motor levels (neurons, muscles, and joints) to simplify motor control. There is much evidence to show that the natural solution to the distribution problem results in highly robust endpoint’s kinematics (Morasso, 1981; Lacquaniti et al., 1983; Flash and Hogan, 1985; Shadmehr and Mussa-Ivaldi, 1994) and kinetics (Hogan, 1985; Mussa-Ivaldi et al., 1985; Flash and Mussa-Ivaldi, 1990; Tsuji et al., 1995) features, called invariant characteristics (Zatsiorsky and Prilutsky, 2012). Motor invariance could provide a clue for understanding the mechanism underlying voluntary movements, because the CNS may impose or exploit these constraints to solve the degrees-of-freedom problem (Bernstein, 1967) essential for motor control.

However, it is an open question: are muscle synergies fundamental primitives or consequences of other primitives? Some researchers have considered motor synergies to be building blocks of movement (d’Avella et al., 2006; Latash, 2008; Cheung et al., 2009; Dominici et al., 2011; Bizzi and Cheung, 2013). However, other researchers have considered that at least some types of motor synergies are not primitives but composites of mechanical impedance (Hogan and Sternad, 2012).

While many aspects of motor control and coordination remain controversial, such as movement reference frame, motor redundancy, and motor primitives (neurological or mechanical origin of motor synergies), our objective in this paper is to provide some evidence supporting the concept that muscle mechanical impedance might provide key insights into unravel motor control intertwined relationships.

In our previous work, we reconsidered muscle synergies from a mechanical engineering aspect and associated them with the reference frame in the muscle space (Uno et al., 2014). The mathematical formulation was theoretically attractive because it suggested that muscle synergies were a function of co-activations by agonist–antagonist (AA) muscle pairs (i.e., composites of mechanical impedance). Moreover, the muscle synergies were viewed as invariant functional modules representing the reference frame in the polar coordinates centered on the specific joint (e.g., shoulder) at the base of the body. Thus, we hypothesized that muscle synergies are consequences of the balance of mechanical impedance, which represents the reference frame in the muscle space.

In this work, we examine our hypothesis from the viewpoint of motor control, learning, and recovery. If muscle synergies are primitives for motor control, learning, and recovery, it would be expected that common synergies are extracted across a variety of different tasks, different subjects, and different motor skills of the subjects. Also, the investigation of muscle synergies for a subject with neuromotor deficits would provide insight into the extent of muscle-synergy invariance, since the fundamental motor functions may be damaged by abnormal muscle tone, which is a common feature after neurological injury. In this study, we tested two experimental paradigms: (1) muscle synergies on motor adaptation and (2) muscle synergies on motor recovery. In our view, muscle synergies strongly relate to mechanical impedance. We also discuss endpoint stiffness and concomitant virtual trajectories in the context of muscle synergies.

## Methods

### Apparatus

**Figure 1:**
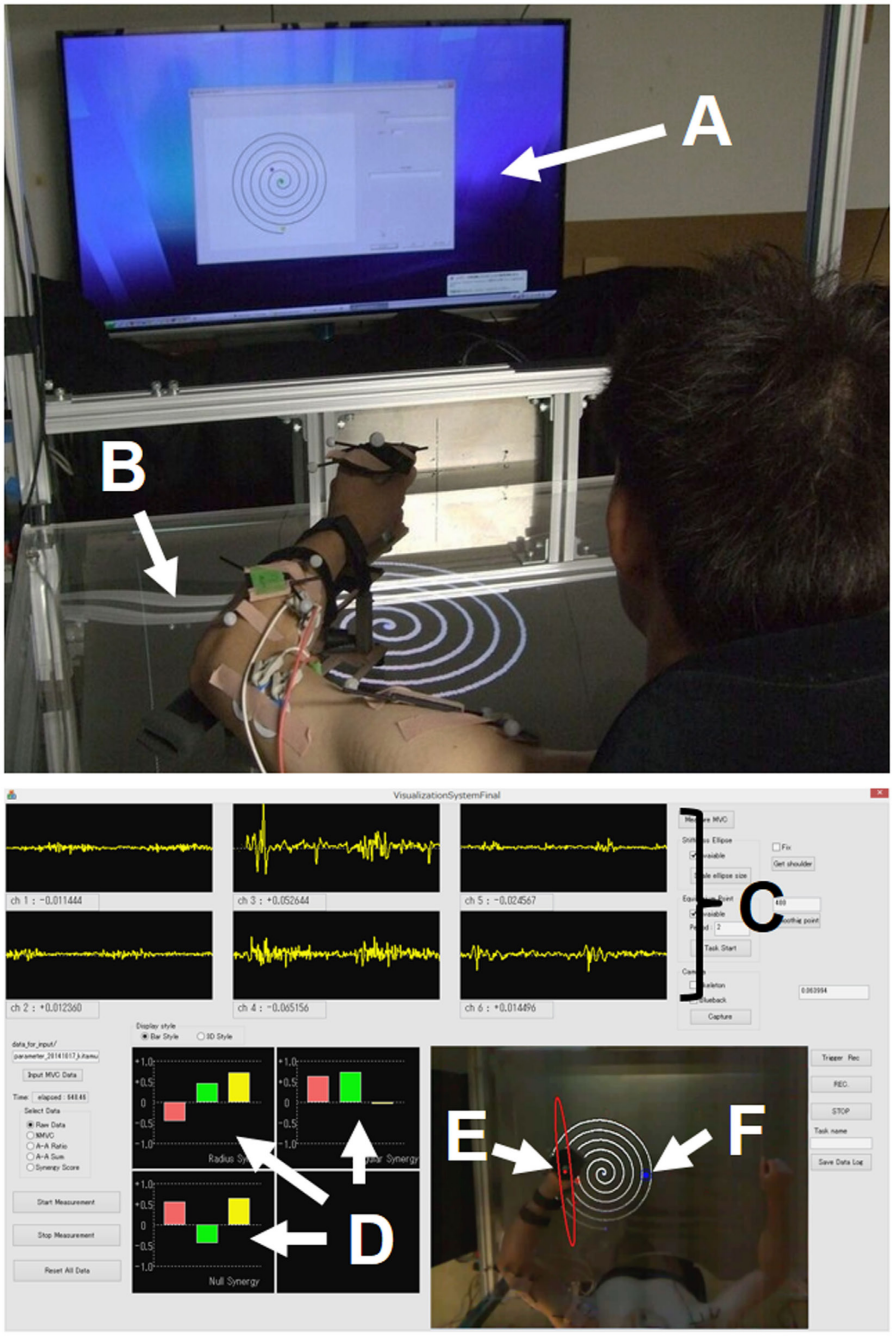
**Experimental setup**. **(A)** Display; **(B)** screen table; **(C)** EMG signals; **(D)** muscle synergies [***u***_R_(***s***) (top left), ***u***_φ_(***s***) (top right), and ***u***_φ×R_(***s***) (bottom left)]; **(E)** endpoint-stiffness ellipse; **(F)** equilibrium point. The subject performed spiral or circle tracing with the non-dominant/dominant hand in a horizontal plane while monitoring a display showing the ideal trajectory. The EMG activities during movements were recorded to analyze muscle synergies, endpoint stiffness, and virtual trajectories.

Multiple muscles in the human neuromuscular system are responsible for coordinating and regulating movement while negotiating within the dynamic environment. The establishment of a systematic framework to explain motor synergies, mechanical impedances, and virtual trajectories is a challenge to the comprehensive understanding of motor control and learning. Assuming that the investigation of multiple muscle activities would lead to a deeper understanding of the neural mechanism underlying voluntary movements, we developed a kinesiological analysis device that enables us to estimate those intrinsic motor characteristics from electromyography (EMG) signals during movement. Figure 1 shows an overview of the system we call the “synergy analyzer*”*. The system consists of a display, a screen table, a chair with harnesses, an arm-support cart with low-friction ball wheels, a motion capture system, and an EMG measurement system.

The subjects sat in the chair with both shoulders fixed in the harnesses and performed voluntary arm movements while looking at the 65-inch display [or a 1.20 m × 0.86 m (width × height) screen table] in front of them. The upper limb was placed on the arm-support cart at shoulder height to eliminate the influence of gravity and restrict arm movement on the horizontal plane. In order to conceptualize the upper limb as a two-link structure, the wrist joint was fixed to the arm-support cart. During the subject’s voluntary arm movements, kinematics and EMG signals were recorded synchronously. Each joint position (left shoulder, right shoulder, left or right elbow, and left or right hand) of the upper limbs was measured using an optical motion capture system with eight cameras (OptiTrack; NaturalPoint, Inc., Corvallis, OR, USA) at 100 Hz.

Electromyography signals of six upper limb muscles [deltoid posterior, deltoid anterior, triceps brachii (long head), biceps brachii, triceps brachii (lateral head), and brachioradialis] were measured with a multi-telemeter system (WEB-5000; Nihon Kohden Corp., Japan) at 1000 Hz. Surface electrodes were attached to the appropriate places on measured muscles as previously described (Criswell, 2010; Perotto, 2011), after cleansing the skin with alcohol (<10 kΩ). The obtained EMG signals were analyzed after the following procedures: bandpass filtering (10–450 Hz), full-wave rectification, smoothing, and normalization to maximum voluntary contraction (MVC), which was reported as percentage of MVC. We followed standard procedure to determine the MVC for each muscle (Hislop and Montgomery, 2007). The synergy analyzer then estimated the muscle synergies, endpoint stiffness, and virtual trajectories from the measured movement data, while superimposing these motor indices in real time (refresh rate, 10 Hz) onto the actual images captured from the top-view camera. The estimation results were provided to the subject on the display (or screen table) for use during biofeedback training.

### Experimental Paradigm

This study focused on the roles of muscle synergies, endpoint stiffness, and virtual trajectories during voluntary training and rehabilitation. To clarify the evolution of these motor characteristics, we performed two experiments.

#### Experiment 1: Motor Adaptation After Training

Eight young subjects (all males, 23 ± 1 years old, right-handed) volunteered for the first experiment. No subject reported any history of neuromuscular disease. The experiment was approved by the Institutional Review Board of Osaka University, and all subjects provided written informed consent before participation.

Each subject performed spiral tracing as fast as possible without touching the lines with his non-dominant (left) hand in a horizontal plane (Figure 1). The maximum radius of the spiral was 21 cm. The visual presentation of the ideal trajectory and current hand position were provided on a display in front of the subject; the ideal spiral trajectory had a spacing of 1.0 cm between lines on the display, which was equivalent to 3.5 cm in the task space. The center of the spiral in the task space was adjusted to correspond to each subject’s hand position in a natural posture. The movement included 5.75 clockwise rotations from outside to inside. To become familiar with the procedure, the subjects performed 20 trials as practice before the first baseline measurement. The subject was then asked to perform the task 50 times per day for 8 days. On the first and last days, the kinematics and EMG signals during the task were measured to analyze the muscle synergies, endpoint stiffness, and virtual trajectories.

#### Experiment 2: Motor Recovery After Rehabilitation

Two elderly subjects, a healthy subject (male, 61 years old, right-handed) and a post-stroke subject (male, 74 years old, right-handed), volunteered for the second experiment. The experiment was approved by the Institutional Review Boards of Osaka University and Senri Chuo Hospital, and both subjects provided informed consent. The healthy subject was a control subject who was of the same generation as the other subject. The post-stroke subject was an acute-stage inpatient with mild-to-moderate right-side hemiplegia but was able to carry out verbal communication. The post-stroke subject performed the experiment twice, before and 2.5 months after rehabilitation.

Each subject performed circle tracing with his dominant (right) hand in a horizontal plane. Because the spiral tracing task was difficult for the post-stroke subject before rehabilitation, we selected a similar trajectory in a smaller circle (radius: 10 cm) so that the task would be easier for him. The post-stroke subject performed the task as fast as possible with his affected hand without any kind of assistance, while the healthy subject performed the task at slow speed (movement time: about 4 sec) to match his movements to those of the post-stroke subject. The kinematics and EMG signals during the task were measured to analyze the muscle synergies, endpoint stiffness, and virtual trajectories.

### Data Analysis

#### AA Ratio and AA Sum

**Figure 2:**
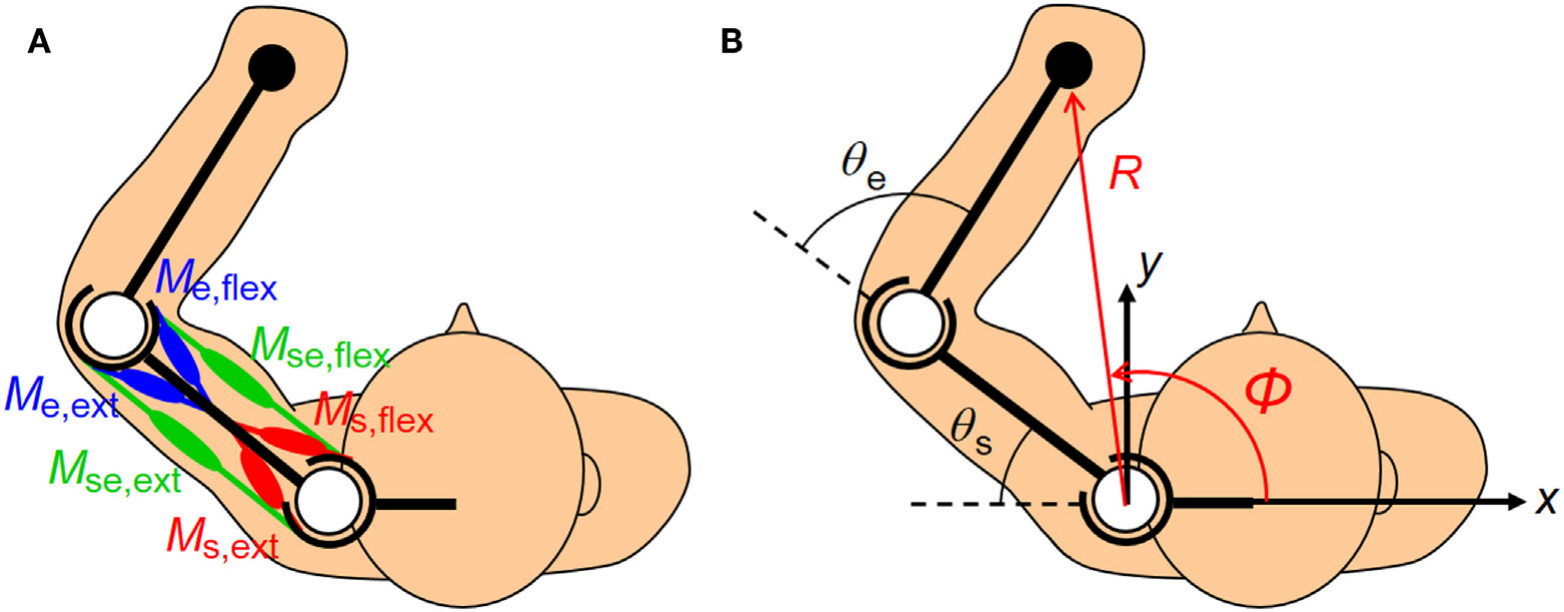
**Human upper limb model**. The human musculoskeletal structure of an upper limb is simplified as a two-link model with six muscles. **(A)** Three pairs of agonist–antagonist muscles are arranged around the shoulder and elbow joints. The paired muscles are indicated in the same color (red, green, and blue). The mono-articular muscle pair around the shoulder joint (*M*_s,ext_ and *M*_s,flex_), bi-articular muscle pair around the shoulder and elbow joints (*M*_se,ext_ and *M*_se,flex_), and mono-articular muscle pair around the elbow joint (*M*_e,ext_ and *M*_e,flex_) are responsible for coordinating and regulating the shoulder and elbow joint movements to control hand movement. **(B)** The hand position in the planar task space can be defined in Cartesian coordinates (*x*, *y*) or polar coordinates (*R*, φ) centered on the shoulder. Note that the polar coordinates (*R*, φ) are defined as positive when the endpoint moves away from the base of the body. These coordinates are the functions of the shoulder and elbow joint angles (θ_s_, θ_e_).

The human upper arm was modeled as a two-link structure with six muscles (Figure 2). We selected the four mono-articular muscles and two bi-articular muscles relevant to the shoulder and elbow movements in a horizontal plane. The chosen muscles were indexed as follows: deltoid posterior (*M*_s,ext_), deltoid anterior (*M*_s,flex_), triceps brachii (long head) (*M*_se,ext_), biceps brachii (*M*_se,flex_), triceps brachii (lateral head) (*M*_e,ext_), and brachioradialis (*M*_e,flex_). These six muscles comprise three pairs of AA muscles. The mono-articular muscle pair around the shoulder joint (*M*_s,ext_ and *M*_s,flex_), bi-articular muscle pair around the shoulder and elbow joints (*M*_se,ext_ and *M*_se,flex_), and mono-articular muscle pair around the elbow joint (*M*_e,ext_ and *M*_e,flex_) are the fundamental functional units for coordinating and regulating the shoulder and elbow joint movements to control hand movement; each muscle pair is composed of two muscles that have opposite (i.e., agonist and antagonist) functions.

To characterize the motor functions of an AA muscle pair around the joint(s), *j*, we defined the following meta-parameters (the AA ratio, *r*_j_, and AA sum, *s*_j_) as the control variables:

(1a) $$r_{\text{j}}=\frac{m_{\text{j},\text{ext}}}{m_{\text{j},\text{ext}}+m_{\text{j},\text{flex}}},\left(j=\text{s},\text{se},\text{e}\right)$$

(1b) $$s_{\text{j}}=m_{\text{j},\text{ext}}+m_{\text{j},\text{flex}},\left(j=\text{s},\text{se},\text{e}\right)$$

where the subscript *j* indicates the joint(s) and corresponds to any one of the shoulder (s), shoulder and elbow (se), and elbow (e) joints; *m*_j,ext_ and *m*_j,flex_ are the EMG activities of the extensor and flexor muscles around the joint(s) *j*. Table 1 lists the motor functions of these AA muscle pairs. The AA ratio contributes to the equilibrium position of the joint angle(s), and the AA sum contributes to the mechanical impedance of the joint(s). Details of the mathematical theory on the AA concept have been previously published (Ariga et al., 2012; Pham et al., 2014; Hirai et al., 2015).

**Table 1:** **Definitions and functions of agonist–antagonist (AA) ratio (r) and AA sum (s)**.

Symbol	Definition	Function
*r*_s_	$\frac{m_{\text{s},\text{ext}}}{m_{\text{s},\text{ext}}+m_{\text{s},\text{flex}}}$	EP control of the shoulder joint angle
*r*_se_	$\frac{m_{\text{se},\text{ext}}}{m_{\text{se},\text{ext}}+m_{\text{se},\text{flex}}}$	EP control of the shoulder and elbow joint angles
*r*_e_	$\frac{m_{\text{e},\text{ext}}}{m_{\text{e},\text{ext}}+m_{\text{e},\text{flex}}}$	EP control of the elbow joint angle
*s*_s_	*m*_s,ext_ + *m*_s,flex_	Stiffness control of the shoulder joint
*s*_se_	*m*_se,ext_ + *m*_se,flex_	Stiffness control of the shoulder and elbow joints
*s*_e_	*m*_e,ext_ + *m*_e,flex_	Stiffness control of the elbow joint

*EP, equilibrium point*.

#### Muscle Synergies

One hypothesis for the spatial and temporal control of limb movements with multiple muscles is the use of hierarchical coordination. In the previous section, we considered the coordination of agonist and antagonist muscles as the lowest level of coordination. This section explains the next level of coordination, intra-limb coordination, and develops the AA concept under the model shown in Figure 2. To derive the relationship among the equilibrium points, AA ratios, and AA sums, we used the following assumptions: (1) each muscle can be described as a spring system whose elastic coefficient and natural length are adjusted according to an EMG signal; (2) the moment arm of each joint is equal and constant; and (3) the lengths of the upper arm (from shoulder joint to elbow joint) and forearm (from elbow to the center of wrist) are equal. In the mathematical sense, the assumption (1) means that the contractile force of a muscle *F*(*m*) can be expressed by

(2) $$F\left(m\right)=K(m)\left(l-l_{0}(m)\right)$$

where *K*(*m*) is the muscle stiffness at EMG activity level *m*, and *l* and *l*_0_(*m*) are the muscle length and natural length of the muscle at EMG activity level *m*. *K*(*m*) and *l*_0_(*m*) are

(3) $$K\left(m\right)=C_{1}(m-C_{2})$$

and

(4) $$l_{0}\left(m\right)=\frac{C_{3}}{K(m)}+C_{4}$$

where *C*_1_, *C*_2_, *C*_3_, and *C*_4_ are constant coefficients that represent the properties of the muscle. The details of our assumption with mathematical formulation have been published previously (Ariga et al., 2012; Hirai et al., 2015). Based on these assumptions, the displacement of the equilibrium-joint angles at the shoulder and elbow, **θ**_**EP**_ = (θ_s,EP_, θ_e,EP_)^T^, can be described with the following equation, using the AA ratios and the AA sums (Pham et al., 2014; Uno et al., 2014; Hirai et al., 2015):

(5) $$\begin{array}{l} \left(\begin{array}{c} \theta_{\text{s},\text{EP}}\\ \theta_{\text{e},\text{EP}} \end{array}\right)=C\left(\begin{array}{ccc} \frac{s_{\text{s}}s_{\text{se}}+s_{\text{s}}s_{\text{e}}}{s_{\text{s}}s_{\text{se}}+s_{\text{e}}s_{\text{se}}+s_{\text{s}}s_{\text{e}}} & \frac{s_{\text{e}}s_{\text{se}}}{s_{\text{s}}s_{\text{se}}+s_{\text{e}}s_{\text{se}}+s_{\text{s}}s_{\text{e}}} & \frac{-s_{\text{e}}s_{\text{se}}}{s_{\text{s}}s_{\text{se}}+s_{\text{e}}s_{\text{se}}+s_{\text{s}}s_{\text{e}}}\\ \frac{-s_{\text{s}}s_{\text{se}}}{s_{\text{s}}s_{\text{se}}+s_{\text{e}}s_{\text{se}}+s_{\text{s}}s_{\text{e}}} & \frac{s_{\text{s}}s_{\text{se}}}{s_{\text{s}}s_{\text{se}}+s_{\text{e}}s_{\text{se}}+s_{\text{s}}s_{\text{e}}} & \frac{s_{\text{e}}s_{\text{se}}+s_{\text{s}}s_{\text{e}}}{s_{\text{s}}s_{\text{se}}+s_{\text{e}}s_{\text{se}}+s_{\text{s}}s_{\text{e}}} \end{array}\right)\times \left(\begin{array}{c} r_{\text{s}}-\frac{1}{2}\\ r_{\text{se}}-\frac{1}{2}\\ r_{\text{e}}-\frac{1}{2} \end{array}\right)\\ \;\theta_{\text{EP}}=C\left(\begin{array}{c} {\text{q}}_{\text{s}}^{\text{T}}\left(s\right)\\ {\text{q}}_{\text{e}}^{\text{T}}\left(s\right) \end{array}\right)\left(\text{r}-\frac{1}{2}\right) \end{array}$$

where *C* is the coefficient determined by the muscle characteristics and the moment arm, ***r*** is the AA ratio vector (*r*_s_, *r*_se_, *r*_e_)^T^, ***s*** is the AA sum vector (*s*_s_, *s*_se_, *s*_e_)^T^, and ***q***_s_(***s***) and ***q***_e_(***s***) are defined as follows:

(6a) $${\text{q}}_{\text{s}}\left(s\right)=\frac{1}{s_{\text{s}}s_{\text{se}}+s_{\text{e}}s_{\text{se}}+s_{\text{s}}s_{\text{e}}}{\left(s_{\text{s}}s_{\text{se}}+s_{\text{s}}s_{\text{e}},s_{\text{e}}s_{\text{se}},-s_{\text{e}}s_{\text{se}}\right)}^{\text{T}}$$

(6b) $$q_{\text{e}}\left(s\right)=\frac{1}{s_{\text{s}}s_{\text{se}}+s_{\text{e}}s_{\text{se}}+s_{\text{s}}s_{\text{e}}}{\left(-s_{\text{s}}s_{\text{se}},s_{\text{s}}s_{\text{se}},s_{\text{e}}s_{\text{se}}+s_{\text{s}}s_{\text{e}}\right)}^{\text{T}}$$

Note that ***q***_s_(***s***) and ***q***_e_(***s***) are composed of the AA sum only. As shown in equation (5), the AA ratio controls the equilibrium-joint angle linearly if ***q***_s_(***s***) and ***q***_e_(***s***) satisfy the condition of being a constant. However, one problem is motor redundancy: the dimension of the AA ratio space always exceeds the dimension of the joint space. The synergy hypothesis emphasizes the use of coordination in solving this ill-posed problem (Bernstein, 1967). We used this hypothesis to introduce a method for extracting the muscle synergies from the human musculoskeletal model. The essence of this technique is that the EP at the endpoint is described based on the polar coordinates system centered on the shoulder (Figure 2). The kinematics of the two degrees-of-freedom arm with the shoulder angle, θ_s_, and elbow angle, θ_e_, determine the unique endpoint position, ***p*** = (*R*, φ)^T^, in the polar coordinates:

(7) $$p=\left(\begin{array}{c} R\\ \phi \end{array}\right)=\left(\begin{array}{c} 2L\text{cos}\frac{\left|\theta_{\text{e}}\right|}{2}\\ \pi -(\theta_{\text{s}}+\frac{\theta_{\text{e}}}{2}) \end{array}\right)$$

where *L* is the length of the upper arm and forearm. By considering a small deviation of ***p*** and substituting Eq. (5) into Eq. (7), we can obtain the relationships among the endpoint EP, AA ratios, and AA sums:

(8) $$\begin{array}{l} \Delta p_{\text{EP}}=J_{\text{R}\phi }(\theta )\cdot \Delta \theta_{\text{EP}}\\ \;=\left(\begin{array}{cc} C_{\text{R}}\left(\theta_{\text{e}}\right) & 0\\ 0 & C_{\phi } \end{array}\right)\left(\begin{array}{c} q_{\text{e}}^{\text{T}}\left(s\right)\\ {\left(q_{\text{s}}\left(s\right)+\frac{q_{\text{e}}\left(s\right)}{2}\right)}^{\text{T}} \end{array}\right)\left(\begin{array}{c} \Delta {\text{r}}_{\text{s}}\\ \Delta {\text{r}}_{\text{se}}\\ \Delta {\text{r}}_{\text{e}} \end{array}\right)\\ \;\approx \left(\begin{array}{cc} C_{\text{R}} & 0\\ 0 & C_{\phi } \end{array}\right)\left(\begin{array}{c} q_{\text{e}}^{\text{T}}\left(s\right)\\ {\left(q_{\text{s}}\left(s\right)+\frac{q_{\text{e}}\left(s\right)}{2}\right)}^{\text{T}} \end{array}\right)\left(\begin{array}{c} \Delta {\text{r}}_{\text{s}}\\ \Delta {\text{r}}_{\text{se}}\\ \Delta {\text{r}}_{\text{e}} \end{array}\right) \end{array}$$

where $J_{\text{R}\phi }(\theta )\left(=\frac{\partial {\left(R,\phi \right)}^{\text{T}}}{\partial (\theta_{\text{s}},\theta_{\text{e}})}\right)$ is a Jacobian matrix that relates the joint space to the task space described in the polar coordinates; *C*_R_(θ_e_) and *C*_φ_ are coefficients determined by the muscle ­characteristics, moment arm of each joint, and upper arm/forearm length *L*. Moreover, $C_{\text{R}}\left(\theta_{\text{e}}\right)\left(=C_{\phi }L\text{sin}\frac{\left|\theta_{\text{e}}\right|}{2}\right)$ can be approximated as a constant $C_{\text{R}}\left(=C_{\text{R}}({\bar{\theta }}_{\text{e}})\right)$ when the elbow is flexed enough during the movement, where ${\bar{\theta }}_{\text{e}}$ is the mean angle of the elbow joint. A remarkable feature of our method is that the formulation is based on the polar coordinates. Owing to the good linear approximation between the task space described in the polar coordinates and the joint space (Mitsuda et al., 1997), the above equation is satisfied in a relatively broad range of work space. Equation (8) indicates that the displacement of the endpoint EP in the polar coordinates can be estimated by projecting the three-dimensional AA ratio vector Δ*r*[=(Δ*r*_s_, Δ*r*_se_, Δ*r*_e_)^T^] onto the two-dimensional subspace composed of *C*_R_***q***_e_(***s***) and $C_{\phi }\left(q_{\text{s}}(s)+\frac{q_{\text{e}}\left(s\right)}{2}\right)$. Based on this informative relationship, we defined the muscle-synergy vectors as

(9a) $$u_{\text{R}}\left(s\right)=\frac{q_{\text{e}}(s)}{\left|q_{\text{e}}(s)\right|}$$

(9b) $$u_{\phi }\left(s\right)=\frac{q_{\text{s}}(s)+\frac{q_{\text{e}}\left(s\right)}{2}}{\left|q_{\text{s}}(s)+\frac{q_{\text{e}}\left(s\right)}{2}\right|}$$

(9c) $$u_{\text{R}\times \phi }\left(s\right)=\frac{u_{\text{R}}(s)\times u_{\phi }(s)}{\left|u_{\text{R}}(s)\times u_{\phi }(s)\right|}$$

where ***u***_R_(***s***) and ***u***_φ_(***s***) indicate the unit vectors for the distributions of the AA ratio vector in the radial and tangential directions, and ***u***_R×φ_(***s***) is defined as the unit vector in the null direction (i.e., the zero space). Muscle synergy in the null direction is not considered to directly contribute to the movement of the endpoint EP but is felt to regulate the endpoint stiffness (Uno et al., 2014). These synergy vectors are the bases for the endpoint EP’s movement in the radial, tangential, and null directions. Note that muscle synergy is a function of the AA sum only. In our definition, the muscle synergy represents the balance of mechanical impedance by co-activations of AA muscles and plays a role as the reference frame in the muscle space for the endpoint EP movement. It is worth noting that muscle synergy becomes constant when ***q***_s_(***s***) and ***q***_e_(***s***) satisfy the condition of being constants. This assumption is not trivial, but the validity of this assumption (i.e., muscle-synergy invariance) is confirmed in the later sections.

#### Endpoint Stiffness

Endpoint stiffness is another index of mechanical impedance, while muscle synergy indicates the balance of mechanical impedance by co-activations of the AA muscles. Assuming a linear relationship between single muscle activation and the corresponding muscle stiffness, the joint stiffness ***K***_j_(***s***) in the static condition can be expressed as the following function of AA sums:

(10) $$K_{\text{j}}(s)=k_{\text{j}}\left(\begin{array}{cc} s_{\text{s}}+s_{\text{se}} & s_{\text{se}}\\ s_{\text{se}} & s_{\text{e}}+s_{\text{se}} \end{array}\right)$$

where *k*_j_ N·m/rad is a gain constant to convert the AA sums to joint stiffness. Under dynamic conditions, such as the presence of a force load, an additional term depending on the hand position and hand force is required (McIntyre et al., 1996). However, we ignored this effect for simplicity, assuming that hand force was minimal in our task. Then, endpoint stiffness ***K***_e_(***s***, **θ**) can be obtained as follows:

(11) $$K_{\text{e}}(s,\theta )={\left(J_{\text{xy}}^{\text{T}}(\theta )\right)}^{-1}\cdot K_{\text{j}}(s)\cdot J_{\text{xy}}^{-1}(\theta )$$

where $J_{\text{xy}}(\theta )\left(=\frac{\partial {\left(x,y\right)}^{\text{T}}}{\partial \left(\theta_{\text{s}},\theta_{\text{e}}\right)}\right)$ is the Jacobian matrix that associates the joint space with the task space in Cartesian coordinates. The endpoint-stiffness matrix can be graphically represented as a stiffness ellipse calculated based on the eigenvalues and eigenvectors of the matrix (Hogan, 1985; Mussa-Ivaldi et al., 1985; Flash and Mussa-Ivaldi, 1990).

#### Virtual Trajectories

By projecting the deviation vector of the AA ratio onto the muscle-synergy vectors, we can obtain the change in the EP at the endpoint. We defined the deviation of synergy activation coefficients (Δ*w*_R_, Δ*w*_φ_, and Δ*w*_R×φ_) as the inner products of the muscle-synergy vectors [***u***_R_(***s***), ***u***_φ_(***s***), and ***u***_R×φ_(***s***)] and the deviation vector of the AA ratio $\Delta r(=r-\bar{r})$, where $\bar{r}$ is the AA ratio at the basis position.

(12a) $$\Delta w_{\text{R}}=u_{\text{R}}^{\text{T}}(s)\cdot \Delta r=u_{\text{R}}^{\text{T}}(s)\cdot (r-\bar{r})$$

(12b) $$\Delta w_{\phi }=u_{\phi }^{\text{T}}(s)\cdot \Delta r=u_{\phi }^{\text{T}}(s)\cdot \left(r-\bar{r}\right)$$

(12c) $$\Delta w_{\text{R}\times \phi }=u_{\text{R}\times \phi }^{\text{T}}(s)\cdot \Delta r=u_{\text{R}\times \phi }^{\text{T}}(s)\cdot (r-\bar{r})$$

The deviation of the endpoint EP is then expressed as

(13) $$\left(\begin{array}{c} \Delta R_{\text{EP}}\\ \Delta \phi_{\text{EP}} \end{array}\right)=\left(\begin{array}{c} \alpha_{\text{R}}\Delta w_{\text{R}}\\ \alpha_{\phi }\Delta w_{\phi } \end{array}\right)$$

where α_R_ and α_φ_ are the gain constants to adjust the scale of the muscle-synergy activation coefficients to the scale of the virtual trajectory, and α_R_ and α_φ_ correspond to *C*_R_ and *C*_φ_ in Eq. (8). The displacement of the endpoint EP in the polar coordinates, ***p***_EP_ = (*R*_EP_, φ_EP_)^T^, can be calculated from a linear combination of muscle-synergy activation coefficients as

(14) $$p_{\text{EP}}=\left(\begin{array}{c} R_{\text{EP}}\\ \phi_{\text{EP}} \end{array}\right)=\left(\begin{array}{c} {\bar{R}}_{\text{EP}}+\Delta R_{\text{EP}}\\ {\bar{\phi }}_{\text{EP}}+\Delta \phi_{\text{EP}} \end{array}\right)=\left(\begin{array}{c} {\bar{R}}_{\text{EP}}+\alpha_{\text{R}}\Delta w_{\text{R}}\\ {\bar{\phi }}_{\text{EP}}+\alpha_{\phi }\Delta w_{\phi } \end{array}\right)$$

where ${\bar{R}}_{\text{EP}}$ and ${\bar{\phi }}_{\text{EP}}$ are the polar coordinates of the endpoint EP at the basis position. In the rest condition at the basis position, we assumed that the actual position and EP position at the endpoint became equal. Finally, the endpoint EP in the Cartesian coordinates can be obtained by the following transformation:

(15) $$\left(\begin{array}{c} x_{\text{EP}}\\ y_{\text{EP}} \end{array}\right)=\left(\begin{array}{c} R_{\text{EP}}\text{cos}\phi_{\text{EP}}\\ R_{\text{EP}}\text{sin}\phi_{\text{EP}} \end{array}\right)$$

The virtual trajectory is a time series and is a succession of EPs at the endpoint. The EP can be represented as a point in the configuration space of muscle synergies, and the virtual trajectory can be identified by tracking the point over time in the muscle-synergy space. Reference control based on EPs or virtual trajectories, that is to say, the EP hypothesis (Feldman, 1966, 1986; Feldman et al., 1990; Feldman and Latash, 2005), has been an influential hypothesis for motor control. Our formulation may give an insight to unify the different ideas of muscle synergies, endpoint stiffness, and virtual trajectories.

## Results

### Experiment 1: Synergy Analysis of Motor Adaptation

The spiral test is a reliable measure of accuracy and speed in upper limb movements; it is usually used in rehabilitation as a qualitative assessment to provide feedback to patients with coordination disorders, such as cerebellar ataxia or Parkinson’s disease (Verkerk et al., 1990). We adopted this measure as an index to reflect the evolution of movement in the non-dominant hand through voluntary training, although the subjects were neurologically and physically intact. The subjects were scored on the time spent to complete the task, with a penalty time added for touching or crossing the lines; the score was defined as the sum of the time spent (from start to goal), the number of times the spiral line was touched multiplied by 3, and the number of times the spiral line was crossed multiplied by 5. The kinematics-assessment score greatly improved for the eight subjects through 8 days of training. The average score for all subjects was 62.1 ± 23.3 (mean ± SD) on the first day and 23.3 ± 11.8 on the last day, respectively, indicating the enhancement of motor performance.

**Figure 3:**
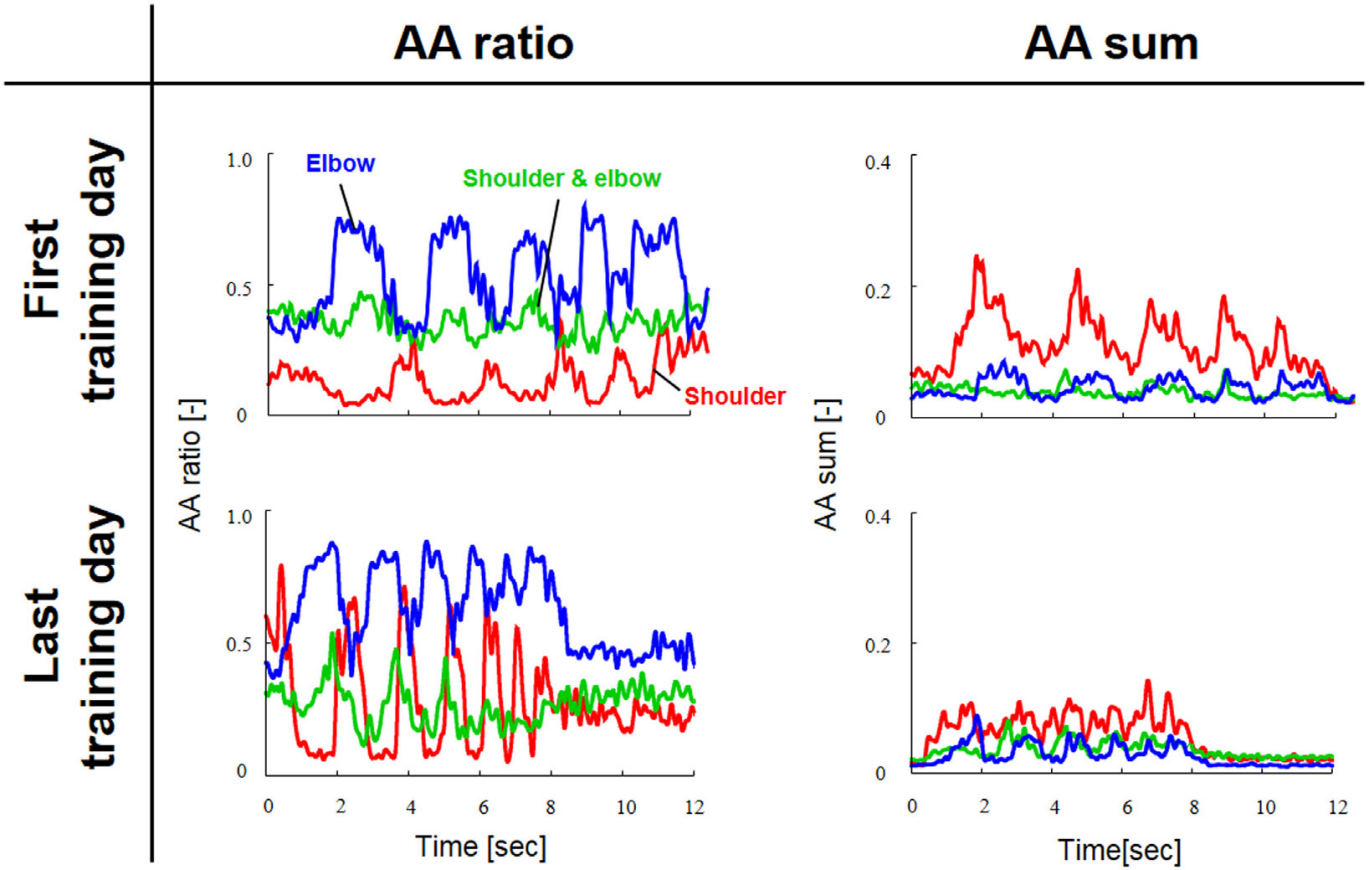
**Typical change in agonist–antagonist (AA) ratios and AA sums from the first and last days of voluntary training (Subject #1)**. The red, green, and blue lines indicate the change in the time-dependent explanatory variable for the AA muscles around the shoulder, shoulder and elbow, and elbow joints, respectively. The AA ratio and AA sum oscillated over time in accordance with rhythmic limb movement. The changes in the AA ratio and AA sum indicate that the voluntary training resulted in a change in the control of EP and stiffness around each joint.

Figure 3 shows a typical AA ratio and AA sum before and after training for one subject (Subject #1). The AA ratio is an explanatory variable ranging from 0 to 1, and the AA sum is an explanatory variable ranging from 0 to 2. The AA ratio and AA sum indicate the degree of extension of the equilibrium-joint angles and of the increase of joint stiffness, respectively. Note that both variables vary with time because they are calculated from EMG signals during movement.

**Figure 4:**
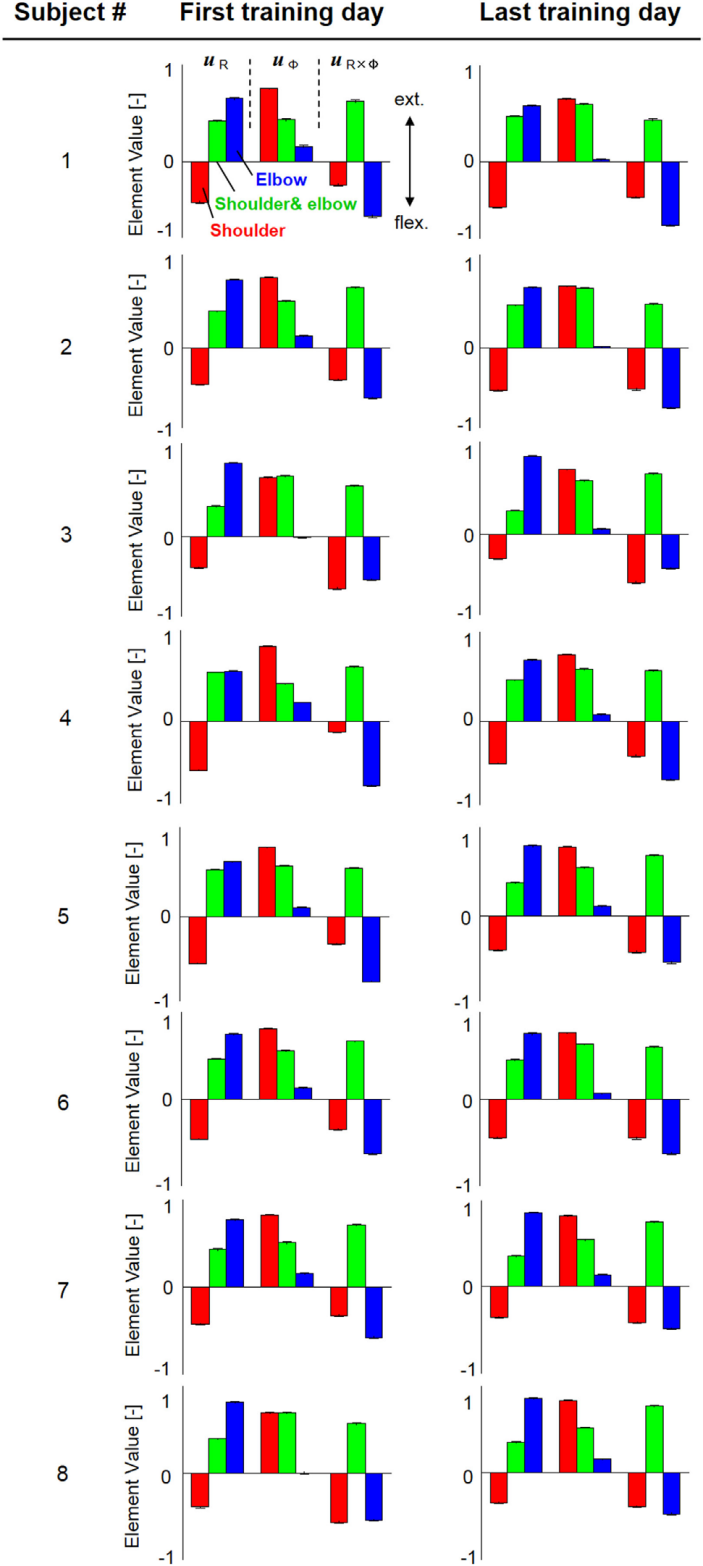
**Muscle synergies from the first and last days of voluntary training for eight subjects**. The three-bar sets represent the muscle synergies in the radial direction (***u***_R_), tangential direction (***u***_φ_), and null direction (***u***_R×φ_). Each colored bar in the muscle synergy indicates the contribution of agonist–antagonist muscle activities to the shoulder (red), shoulder and elbow (green), and elbow joint (blue) movement. Muscle synergies exhibited similar patterns regarding both intra- and inter-individual variations during training, demonstrating the existence of common and invariant reference frames for motor representation that are independent of the level of motor learning.

Figure 4 shows the change in muscle synergies for the eight subjects before and after training, using the method described in the previous section. In each graph, the left, central, and right groups of the three-bar set (red, green, and blue) illustrate the muscle synergy in the radial direction [***u***_R_(***s***)], tangential direction [***u***_φ_(***s***)], and null direction [***u***_R×φ_(***s***)], respectively. The three colored bars in each muscle synergy represent the element values of the muscle-synergy vector, and each value quantifies the contribution of AA muscle activities to the shoulder, shoulder and elbow, and elbow joint movement, respectively. For a summary of the mean changes and SDs of muscle synergies, see Table 2. Table 3 illustrates the inner-product (IP) values between muscle-synergy vectors computed from EMG signals in Experiment 1, indicating the similarity of muscle synergies in both inter-individual and intra-individual variations.

**Table 2:** **Element values of muscle synergies before and after voluntary training on spiral tracing (A) first training day and (B) last training day**.

Subject #	*u*_R_(*s*)			*u*_**φ**_(*s*)			*u*_R**×φ**_(*s*)		
	Shoulder	Shoulder and elbow	Elbow	Shoulder	Shoulder and elbow	Elbow	Shoulder	Shoulder and elbow	Elbow
**(A) First training day**									
1	−0.471 (±0.002)	0.471 (±0.002)	0.730 (±0.003)	0.844 (±0.001)	0.488 (±0.003)	0.178 (±0.002)	−0.270 (±0.003)	0.699 (±0.003)	−0.629 (±0.003)
2	−0.425 (±0.002)	0.425 (±0.002)	0.789 (±0.002)	0.816 (±0.002)	0.543 (±0.003)	0.136 (±0.002)	−0.370 (±0.003)	0.701 (±0.003)	−0.579 (±0.003)
3	−0.355 (±0.002)	0.355 (±0.002)	0.856 (±0.002)	0.689 (±0.003)	0.701 (±0.003)	−0.006 (±0.003)	−0.601 (±0.004)	0.588 (±0.003)	−0.494 (±0.003)
4	−0.569 (±0.001)	0.569 (±0.001)	0.581 (±0.002)	0.871 (±0.000)	0.434 (±0.001)	0.218 (±0.001)	−0.125 (±0.001)	0.630 (±0.002)	−0.744 (±0.002)
5	−0.541 (±0.001)	0.541 (±0.001)	0.632 (±0.002)	0.796 (±0.001)	0.586 (±0.002)	0.105 (±0.001)	−0.314 (±0.002)	0.560 (±0.002)	−0.748 (±0.002)
6	−0.461 (±0.002)	0.461 (±0.002)	0.746 (±0.002)	0.811 (±0.001)	0.554 (±0.002)	0.129 (±0.002)	−0.354 (±0.003)	0.664 (±0.002)	−0.628 (±0.002)
7	−0.432 (±0.002)	0.432 (±0.002)	0.780 (±0.003)	0.831 (±0.002)	0.513 (±0.003)	0.159 (±0.003)	−0.330 (±0.003)	0.715 (±0.003)	−0.582 (±0.004)
8	−0.393 (±0.002)	0.393 (±0.002)	0.818 (±0.003)	0.694 (±0.003)	0.695 (±0.004)	−0.001 (±0.004)	−0.568 (±0.004)	0.567 (±0.004)	−0.547 (±0.004)
**(B) Last training day**									
1	−0.528 (±0.002)	0.528 (±0.002)	0.647 (±0.003)	0.726 (±0.002)	0.664 (±0.003)	0.031 (±0.003)	−0.413 (±0.004)	0.486 (±0.004)	−0.735 (±0.003)
2	−0.492 (±0.002)	0.492 (±0.002)	0.700 (±0.003)	0.714 (±0.001)	0.692 (±0.002)	0.011 (±0.002)	−0.480 (±0.003)	0.505 (±0.002)	−0.692 (±0.003)
3	−0.281 (±0.002)	0.281 (±0.002)	0.910 (±0.001)	0.754 (±0.002)	0.628 (±0.003)	0.063 (±0.003)	−0.553 (±0.004)	0.705 (±0.003)	−0.388 (±0.003)
4	−0.482 (±0.002)	0.482 (±0.002)	0.714 (±0.003)	0.777 (±0.002)	0.612 (±0.003)	0.083 (±0.002)	−0.398 (±0.004)	0.594 (±0.003)	−0.669 (±0.003)
5	−0.388 (±0.003)	0.388 (±0.003)	0.813 (±0.003)	0.802 (±0.002)	0.563 (±0.003)	0.119 (±0.003)	−0.411 (±0.004)	0.699 (±0.003)	−0.530 (±0.005)
6	−0.449 (±0.003)	0.449 (±0.003)	0.753 (±0.003)	0.763 (±0.001)	0.634 (±0.002)	0.065 (±0.002)	−0.449 (±0.003)	0.603 (±0.003)	−0.635 (±0.004)
7	−0.355 (±0.002)	0.355 (±0.002)	0.856 (±0.002)	0.821 (±0.001)	0.544 (±0.003)	0.138 (±0.002)	−0.418 (±0.003)	0.753 (±0.003)	−0.486 (±0.003)
8	−0.351 (±0.003)	0.351 (±0.003)	0.856 (±0.002)	0.833 (±0.002)	0.522 (±0.003)	0.155 (±0.002)	−0.394 (±0.003)	0.770 (±0.003)	−0.477 (±0.004)

**Table 3:** **Inner-product values between muscle synergies (experiment 1)**.

	*u*_R_(*s*)	*u*_φ_(*s*)	*u*_R×φ_(*s*)
Inter-individual variations (between first and last training days)	0.968 (±0.015)	0.963 (±0.017)	0.933 (±0.018)
Intra-individual variations (first training day)	0.963 (±0.021)	0.959 (±0.025)	0.923 (±0.036)
Intra-individual variations (last training day)	0.953 (±0.023)	0.974 (±0.009)	0.931 (±0.028)

**Figure 5:**
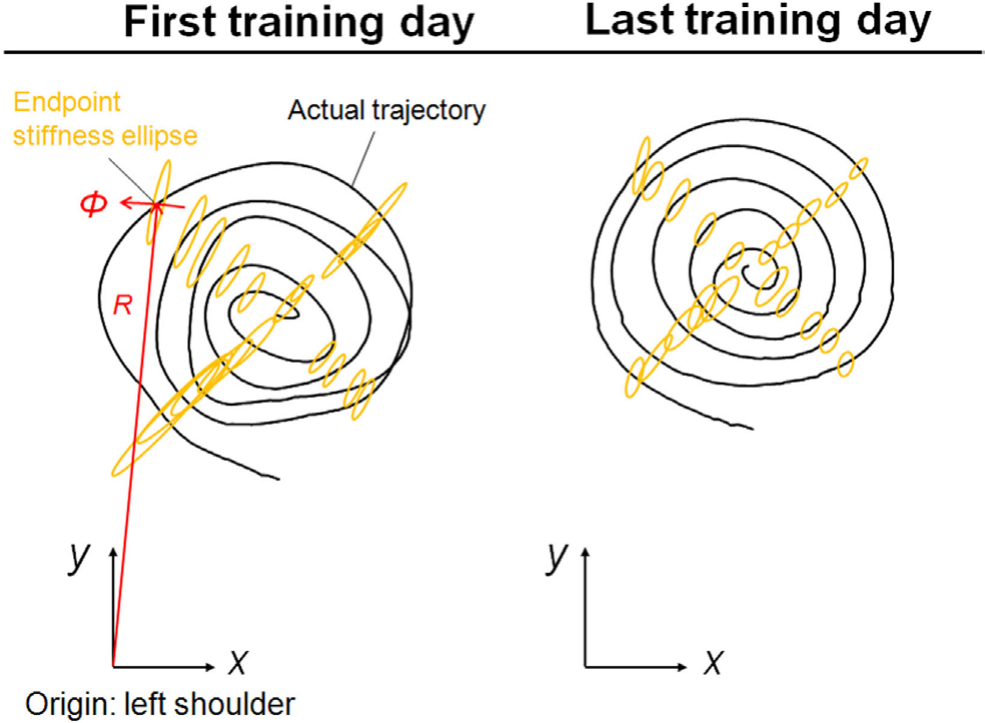
**Typical change in endpoint stiffness from the first and last days of voluntary training (Subject #1)**. While its size changed, the shape and orientation of the endpoint-stiffness ellipse did not alter much during training. The orientation of the major axis of the ellipse tended to tilt toward the direction connecting the shoulder and the endpoint (i.e., the radial direction). This indicates that the endpoint stiffness in the tangential direction tends to be far smaller than that in the radial direction.

**Figure 6:**
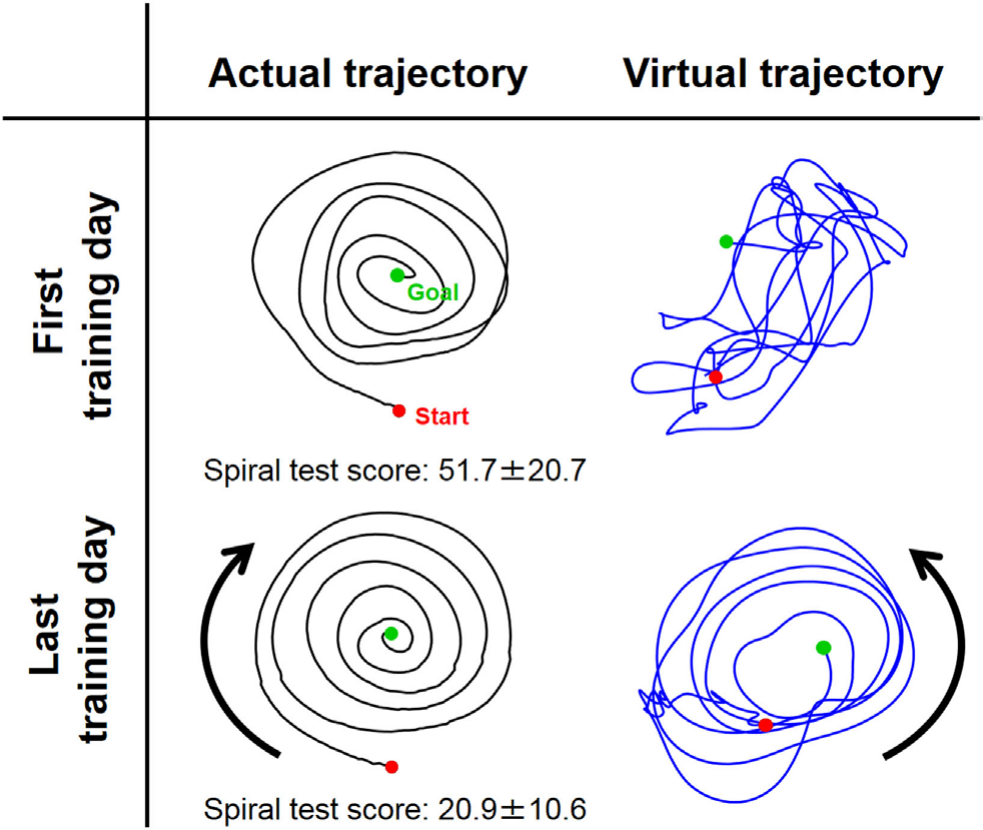
**Typical change in virtual trajectory from the first and last days of voluntary training (Subject #1)**. The change in the virtual trajectories (blue) was extreme compared to that for the actual trajectories (black). The virtual trajectories were organized from disordered patterns into well-regulated but slightly distorted spiral patterns that rotated in the opposite direction of the actual trajectory. The score of the spiral test decreased correspondingly from 51.7 ± 20.7 to 20.9 ± 10.6, indicating that motor performance was improved in terms of speed and accuracy.

**Figure 7:**
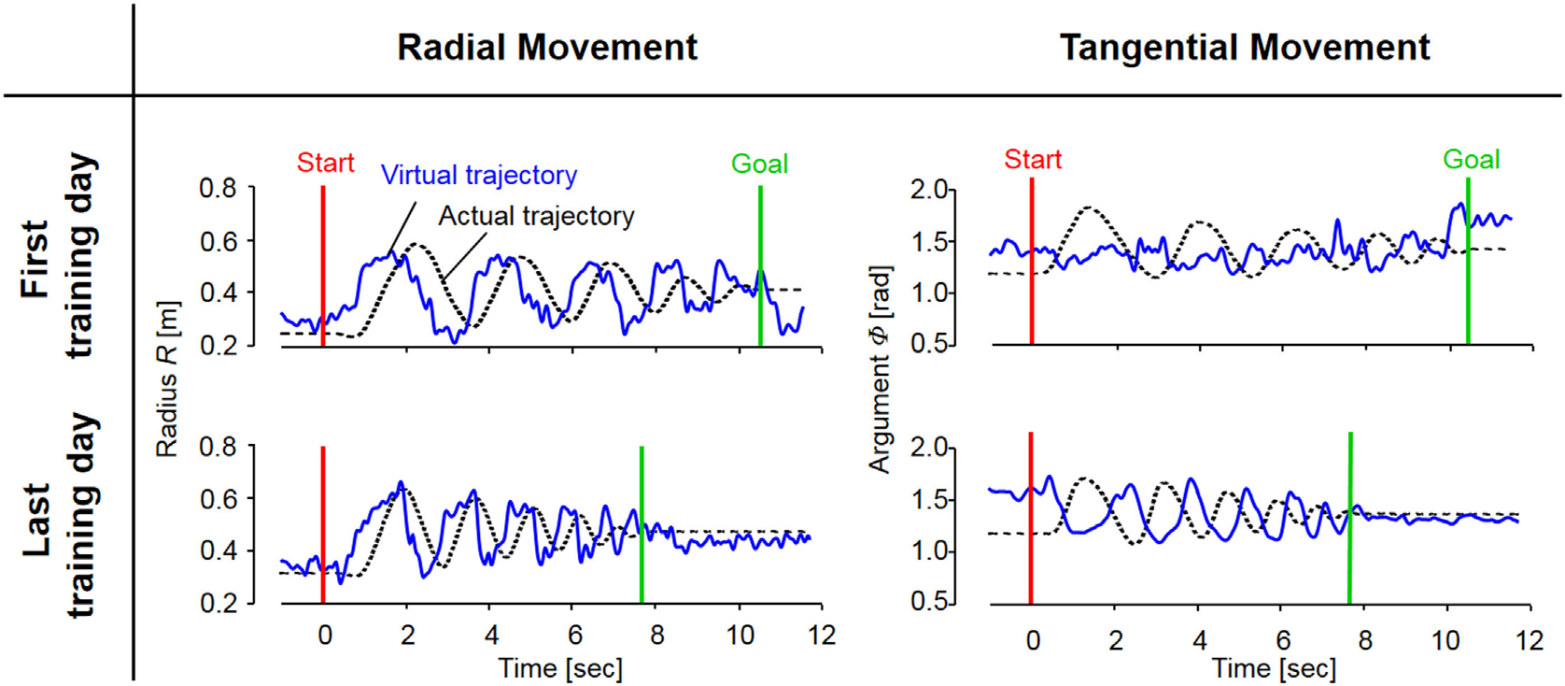
**The evolution of actual and virtual trajectories in the radial and tangential directions (Subject #1)**. The virtual trajectory during fast spiral tracing for Subject #1 improved with voluntary training. In particular, the evolution of the virtual trajectory in the tangential direction was extreme, and it changed into a rhythmic movement that preceded the actual trajectory oscillating almost out of phase; the virtual trajectory in the radial direction developed into a trajectory that preceded the actual trajectory oscillating almost in phase. The oscillation frequencies of the virtual trajectory in both directions became shorter in accordance with those of the actual trajectory. These evolutionary characteristics of the virtual trajectory are representative of those observed for all eight subjects.

Figure 5 shows typical endpoint stiffness before and after voluntary training (the first and last days of training) for Subject #1. The endpoint-stiffness ellipses during movement were compared between corresponding hand positions. Figure 6 shows typical actual and virtual trajectories before and after voluntary training for Subject #1. The red and green circles are the start and goal points, and the arrow indicates the direction in which each trajectory progresses. Figure 7 shows the actual and virtual trajectories in the radial and tangential directions, which correspond to those trajectories in Figure 6. Figure 7 also shows the change in movement time, indicating significant improvement in motor performance. The observed evolution characteristics in these figures are representative of those for all eight subjects.

### Experiment 2: Synergy Analysis of Motor Recovery

The functional independence measure (FIM) score is a widely used scale of disability severity that quantifies the impact of impairment on the performance of daily activities (Granger et al., 1986; Carr and Shepherd, 2010). The FIM score (maximum score: 126) of the post-stroke subject in this study was 44 points before rehabilitation and 67 points after 2.5 months of rehabilitation. These scores indicate that the subject’s motor function improved through therapist-based exercise in rehabilitation. In agreement with the FIM score’s change, the average movement time for the post-stroke subject in our task improved from about 6 s to about 4 s before and after rehabilitation; the average movement time for the healthy subject was about 4 s.

**Figure 8:**
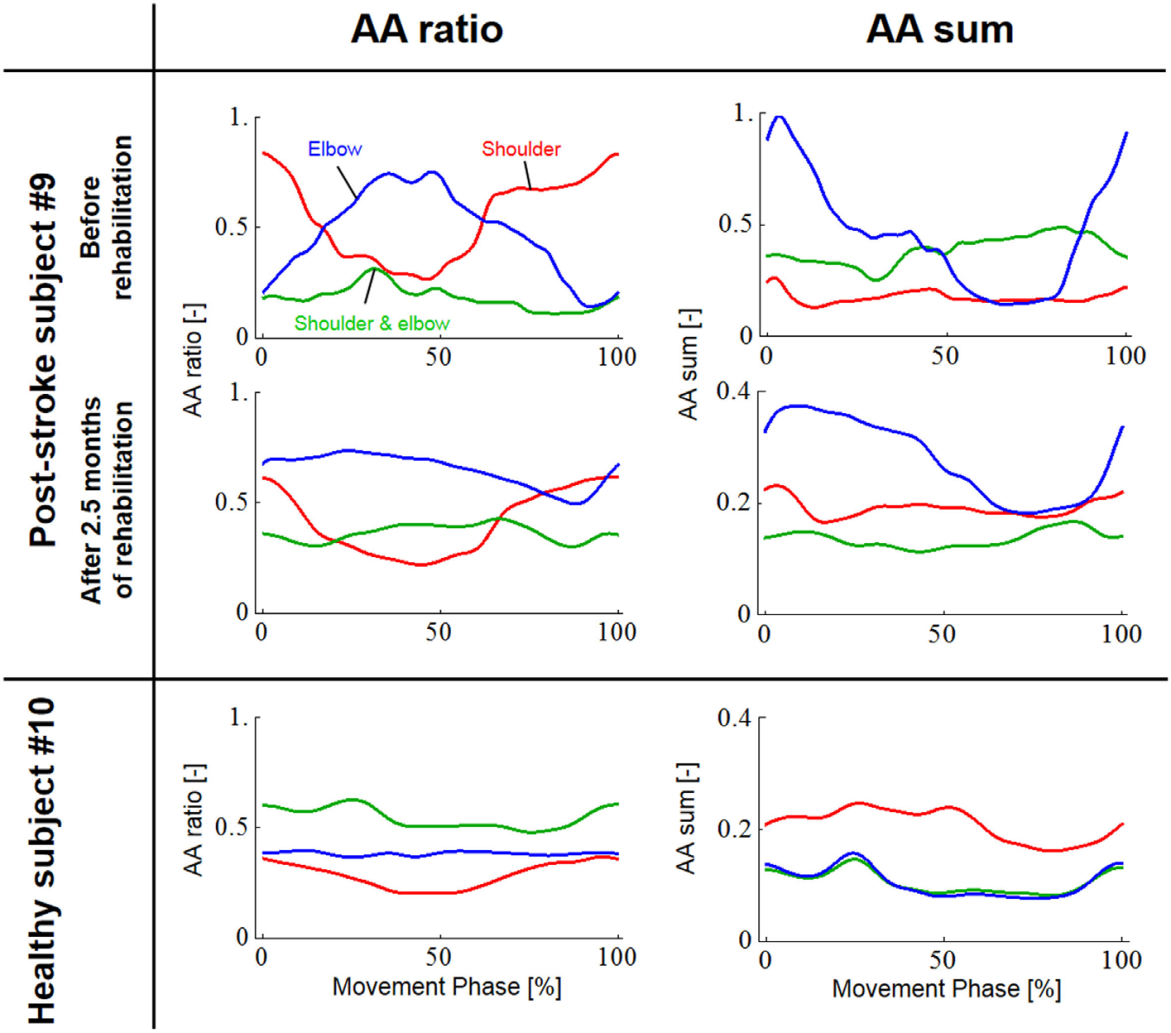
**Change in the agonist–antagonist (AA) ratios and AA sums for the post-stroke subject before and after 2.5 months of rehabilitation**. The red, green, and blue lines indicate the change in the time-dependent explanatory variable for the AA muscles around the shoulder, shoulder and elbow, and elbow joints, respectively. Each data set was normalized with respect to a period of movement time. The changes in the AA ratio and AA sum indicate that rehabilitation resulted in change in the control of EP and stiffness around each joint. In particular, the AA sum was improved although it was still far from the level observed from the healthy subject. (Note the different range of the graphs for AA sums before and after rehabilitation.)

**Figure 9:**
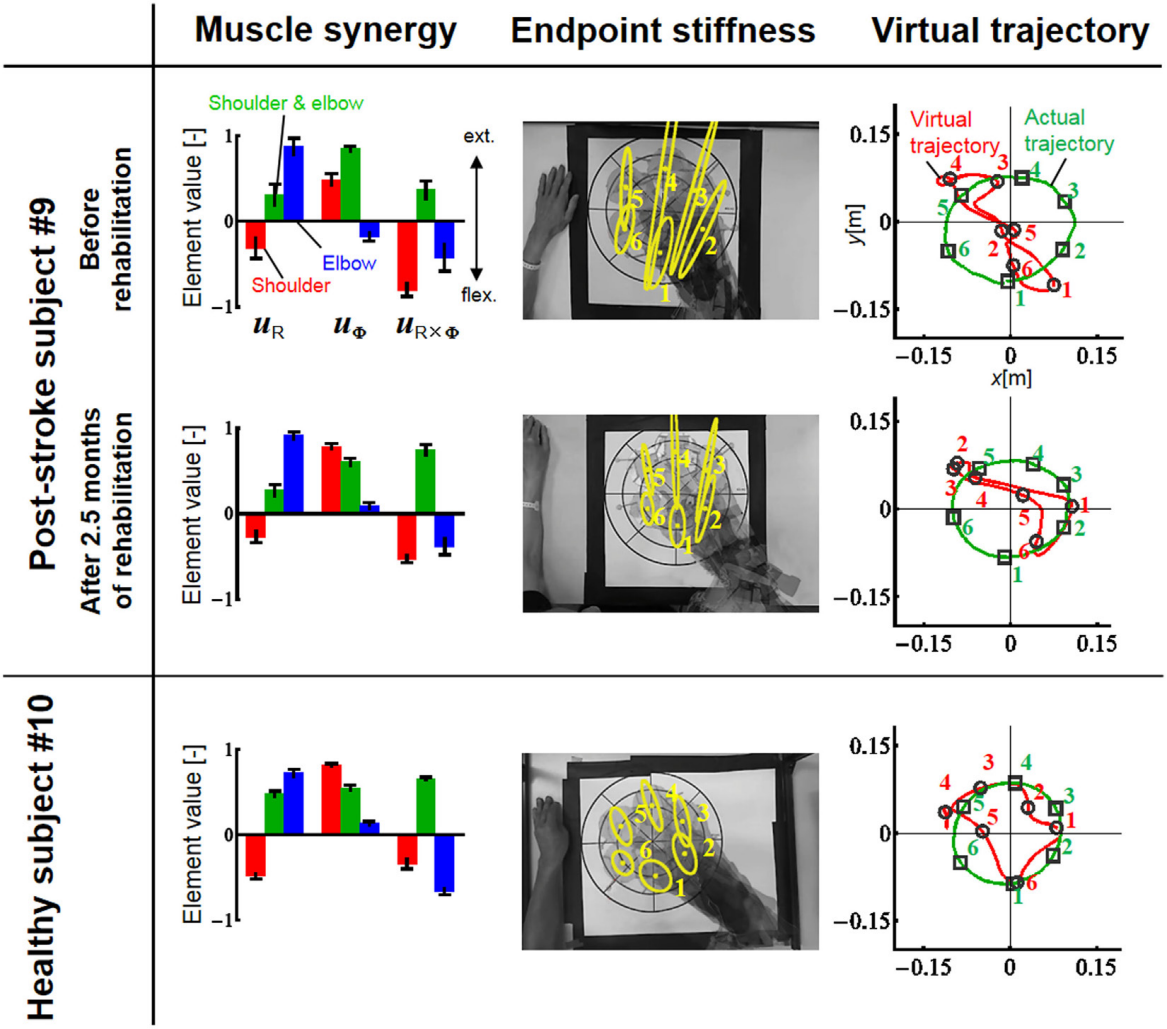
**Change in muscle synergies, endpoint stiffness, and virtual trajectories before and after 2.5 months of rehabilitation**. The top, middle, and bottom blocks are for the post-stroke subject before rehabilitation, after 2.5 months of rehabilitation, and for the healthy subject from the same generation, respectively. The three-bar sets represent the muscle synergies in the radial direction (***u***_R_), tangential direction (***u***_φ_), and null direction (***u***_R×φ_). Each number on the stiffness ellipses indicates the progress of movement with time and corresponds to each number of points on the actual and virtual trajectories.

Since obvious recovery was observed in the post-stroke subject, we then compared the motor indices of muscle synergies, endpoint stiffness, and virtual trajectories, which characterize the coordination and regulation of multiple muscle activities before and after rehabilitation. Figure 8 shows the changes in the AA ratios and AA sums for the post-stroke subject before and after 2.5 months of rehabilitation, as well as the changes in the AA ratios and AA sums for the same-generation healthy subject. Figure 9 shows the changes in muscle synergies, endpoint stiffness, and virtual trajectories, which can be estimated by the proposed algorithm with the AA ratio and AA sum. The actual trajectory was also plotted on the graph of the virtual trajectory in Figure 9 as one of the indices of motor recovery, although significant change was not observed. Details of the mean changes and SDs of muscle synergies are summarized in Table 4. Table 5 compares the IP values between muscle-synergy vectors for different variations in Experiment 2: inter-individual variations, intra-individual variations, and intra-task variations.

**Table 4:** **Element values of muscle synergies for a post-stroke subject before and after rehabilitation and for a healthy elderly subject**.

Subject #	*u*_R_(*s*)			*u*_φ_(*s*)			*u*_R×φ_(*s*)		
	Shoulder	Shoulder and elbow	Elbow	Shoulder	Shoulder and elbow	Elbow	Shoulder	Shoulder and elbow	Elbow
9a (post-stroke subject before rehabilitation)	−0.300 (±0.142)	0.300 (±0.142)	0.875 (±0.117)	0.490 (±0.055)	0.850 (±0.024)	−0.180 (±0.039)	−0.802 (±0.082)	0.376 (±0.101)	−0.403 (±0.192)
9b (post-stroke subject after rehabilitation)	−0.276 (±0.067)	0.276 (±0.067)	0.915 (±0.043)	0.788 (±0.031)	0.606 (±0.045)	0.091 (±0.038)	−0.530 (±0.040)	0.746 (±0.062)	−0.385 (±0.096)
10 (healthy elderly subject)	−0.487 (±0.027)	0.487 (±0.027)	0.723 (±0.036)	0.822 (±0.016)	0.552 (±0.030)	0.134 (±0.023)	−0.334 (±0.054)	0.656 (±0.013)	−0.670 (±0.032)

**Table 5:** **Inner-product values between muscle synergies (experiment 2)**.

	*u*_R_(*s*)	*u*_φ_(*s*)	*u*_R×φ_(*s*)
Inter-individual variations (before and after rehabilitation)	0.966	0.885 (<0.9)	0.861 (<0.9)
Intra-individual variations (between post-stroke subject after rehabilitation and healthy subject)	0.930	0.994	0.924
Intra-task variations (between fast spiral tracing and slow circle tracing)	0.970	0.982	0.952

## Discussion

### Muscle Synergies as Reference Frames in Muscle Space

Focusing on the coordination among activities of AA muscles, here we discuss the relationships among the muscle synergies, endpoint stiffness, and virtual trajectories. To our knowledge, muscle synergy is a coordination index defined as a function of co-activations of AA muscles. It is a composite unit associated with mechanical impedance and is also a functional module representing the reference axis in the polar coordinate system for the displacement of an EP in the task space. In short, muscle synergies represent the reference frame in the muscle space that may be used in motor planning for endpoint control. Endpoint stiffness is another index of mechanical impedance; the balance of the co-activations of AA muscles determines not only the muscle synergies but also the shape and orientation of the endpoint-stiffness ellipse. The virtual trajectory is a time sequence of EPs at the endpoint. The EP can be represented as a point in the configuration space of muscle synergies, and the virtual trajectory can be identified by tracking the point over time in the muscle-synergy space. The mathematical relationships among these inter-winding motors were derived from the physical modeling of the musculoskeletal structure with multiple AA muscles.

### Physics-Based Approach to Muscle-Synergy Extraction

Our approach may provide a new perspective in understanding motor control and learning. Motor synergies are usually extracted by applying statistical techniques to explanatory variables, such as joint angles and EMG signals, which may be the set of motor states resulting from CNS commands based on fewer motor modules. The results of factor decomposition are, however, not necessarily interpretable with such explanatory variables even though the factors successfully reduce the dimensionality of movement. Therefore, the physical meaning of motor synergies is not clear in most cases, in particular, in the case of muscle synergy because EMG includes information on both kinematic and kinetic aspects; muscles work for both joint displacement and joint impedance. EMG is phenomenologically interpreted as an electrical signal originating from the depolarization of the muscle fibers. However, the relationship between muscle activation and movement is not fully understood. We assumed that the CNS controls the equilibrium state and mechanical impedance for multi-joint movements by changing specific neurophysiological parameters (Feldman et al., 1990) and that EMG consequently reflects at least these two pieces of information. Then, the statistical analysis of original EMG signals may result in yielding the makeshift factors, which are usually task-dependent and/or subject-dependent.

To gain insight into the physical meaning of muscle synergies, this study examined the AA concept using the following explanatory variables: the AA ratio, which is related to the equilibrium-joint angle, and the AA sum, which is associated with the joint stiffness. Since the AA concept originates from the control of a robotic system with antagonistic pneumatic artificial muscles, muscle synergy extracted under the AA concept has a clear physical meaning. Similar ideas for the control of AA muscles can be found in the field of neuroscience [e.g., the ratio of the tensions of AA muscles (Lestienne et al., 1981; Bizzi et al., 1984), mechanical impedance and co-activation of AA muscles (Hogan, 1984), and the control of the EP and level of co-contraction for joint movement (Feldman et al., 1990)]. However, our AA concept is strictly different from these. The AA concept can be regarded as another form of the EP hypothesis (Feldman, 1966, 1986; Feldman et al., 1990; Feldman and Latash, 2005) and can be extended to the novel concept of EP-based synergies (Pham et al., 2014; Uno et al., 2014; Hirai et al., 2015).

It is also worth noting that the muscle synergy derived from our approach is composed of AA sums only (see Eqs 9a–c). This formulation means that the muscle synergies themselves are not motor primitives but consequences of modulation of mechanical impedance, which may be one of the motor primitives. We believe that our findings are in line with the idea of dynamic primitives, which Hogan and Sternad recently argued (Hogan and Sternad, 2012). Nevertheless, muscle synergies may play roles as functional modules – that is, a reference frame in muscle space. Muscle synergies represented as the balance of mechanical impedance may be called “kinetic synergies.” Thus, our study is categorized as a physics-based approach and is clearly different from most studies, which are categorized as statistical-based approaches (d’Avella et al., 2006; Cheung et al., 2009, 2012; Dominici et al., 2011; Bizzi and Cheung, 2013; Roh et al., 2013) for extracting muscle synergies. Although we do not discuss null synergy much in this paper, the idea of null synergy, which the statistical approach cannot extract from the data, is informative. The physics-based approach is a powerful way to reverse engineer the control mechanism underlying the neuromusculoskeletal system in the dynamic environment. For more details on muscle synergies based on the AA concept, refer also to our recent publications (Koba et al., 2014; Oku et al., 2014, 2015; Uno et al., 2014).

### Muscle Synergies, Endpoint Stiffness, and Virtual Trajectories in Motor Adaptation

How do muscle synergies or the balance of mechanical impedance affect motor enhancement if they represent a reference frame in muscle space? We measured the similarity of muscle-synergy vectors among subjects before and after voluntary training, based on the IP value of the corresponding two muscle-synergy vectors (Table 3). The results revealed that the muscle synergies before and after training exhibited similar patterns regarding both intra-individual variations and inter-individual variations. It is also notable that the muscle synergies were held almost constant despite being calculated from time-varying AA sums. The SDs of muscle synergies were sufficiently small for all subjects (Figure 4, Table 2). These results demonstrate the invariant kinetic characteristics that the CNS may exploit for movement planning.

It is mathematically evident that the muscle synergies represent the bases of the polar coordinates. The results suggest that the invariant reference frame for motor representation is encoded into time-varying biological signals (Figure 3) and that the reference frame is not only common among normal subjects but also independent of the level of learning. Thus, we hypothesize that the muscle synergies may be functional modules to link the muscle space to the task space and that they may be a coordinate system for motor control. Moreover, the invariance of muscle synergies may be related to the stable characteristics of endpoint stiffness since the muscle synergies represent the balance of mechanical impedance by co-activations of AA muscles. In our task, the shape and orientation of the endpoint-stiffness ellipse did not change much during training; however, its size changed (Figure 5). The orientation of the major axis of the ellipse tended to keep tilting toward the direction connecting the shoulder and the endpoint (i.e., the radial direction). This indicates that endpoint stiffness in the tangential direction always tends to be far smaller than that in the radial direction.

In contrast to these hard-wired characteristics in the CNS, virtual trajectories showed drastic changes with motor enhancement. The virtual trajectories were organized from disordered patterns into smooth spiral patterns that rotated in the opposite direction of the actual trajectories (Figure 6). As shown in Figure 7, in both cases before and after training, the virtual trajectories showed oscillating movements that preceded the actual trajectories with similar sequences showing gradually decreasing amplitudes. However, the different phase relationship between the actual and virtual trajectories emerged in each direction after training. In the radial direction, high endpoint stiffness caused endpoint EP movement with about a 0° phase shift. In the tangential direction, the far smaller endpoint stiffness caused endpoint EP movement with about a 180° phase shift. The coupling of these directional mechanical impedances yielded a counterintuitive observation, i.e., the opposite rotation of virtual trajectories. This phenomenon can be observed in fast movements. The finding indicates that the CNS requires an internal model (Gomi and Kawato, 1997) to achieve dynamic compensation in the process of motor control and learning.

### Muscle Synergies, Endpoint Stiffness, and Virtual Trajectories in Motor Recovery

In contrast to our results from Experiment 1, significant changes (IP < 0.9) were observed in the muscle synergies for the post-stroke subject before and after rehabilitation (Table 5). This observation relates to the disrupted inter-joint coordination commonly observed in arm movements after stroke. In our view, the alteration of muscle synergies indicates a breach in the basis for motor control, and it may influence the reference frame essential for sensorimotor transformation. In the case of the post-stroke subject, the abnormal co-activation of bi-articular AA muscles (green bars in Figure 9) yielded different muscle synergies, especially in the tangential direction. The SDs of abnormal muscle synergies were then within a tolerance (small) level and, thus, the abnormal muscle synergies could also be regarded as the invariant bases for the polar coordinates. In other words, the reference frame in the muscle space was held by other coordinated muscles.

Before rehabilitation, the post-stroke subject may have exploited motor redundancy to regulate multiple muscles in order to manage his impairments following neurological injury, and he may therefore have achieved an invariant coordination different from that of normal subjects. Since the muscle synergies represented a balance of mechanical impedance by co-activation of AA muscles, the changes in muscle synergies significantly affected the endpoint-stiffness characteristics. The endpoint-stiffness ellipse of the post-stroke subject before rehabilitation was elongated, and the orientation of its major axis indicated a more clockwise rotation than that observed in the healthy subject. The primary cause may have been the hypertonicity of the bi-articular muscles; an increase in the co-activation of bi-articular muscles (*s*_se_) tends to modify the eigenvalues and eigenvectors of the joint stiffness matrix ***K***_j_(***s***), enlarging the stiffness ellipse and rotating its major axis in a clockwise direction. These characteristics of the endpoint-stiffness ellipse may explain the typical dysfunctions: flexor synergy (characterized by simultaneous shoulder abduction and elbow flexion) and extensor synergy (characterized by simultaneous shoulder adduction and elbow extension) (Brunnstrom, 1970). Moreover, alteration of muscle synergies results in significant distortion of not only endpoint stiffness but also concomitant virtual trajectories. The virtual trajectory moved within a limited range and tended to move in the fixed direction of the minor axis of the endpoint-stiffness ellipse.

However, rehabilitation caused a fundamental change in motor control in the post-stroke subject. The muscle synergies after rehabilitation exhibited similar patterns (IP > 0.9) to those of the healthy subject (Table 5). This result indicates that the post-stroke subject regained the normal patterns of muscle synergies in the process of recovery. Moreover, these patterns were similar to the patterns extracted from the subjects during the fast-spiral-tracing task (IP > 0.9). This case study provides only preliminary evidence for common muscle synergies across a variety of different tasks, different subjects, and different motor skills of the subjects. The endpoint stiffness also recovered, along with the recovery of the corresponding muscle synergies. The shape of the endpoint-stiffness ellipse was shortened, and the orientation of the major axis rotated slightly counter-clockwise. These results provide evidence that the post-stroke subject was on the course of recovery. This analysis was based on data after 2.5 months of rehabilitation, and it is supposed that the post-stroke subject has the potential to recover motor function with further rehabilitation. Interestingly, in the process of recovery, the muscle synergies regained normal patterns earlier than did the virtual trajectories. The virtual trajectory did not fully recover after 2.5 months of rehabilitation. This indicates that the muscle synergies playing a role as the reference frame in the muscle space are fundamental for motor control. This is a reasonable conclusion because the virtual trajectories are defined by the configuration space of muscle synergies.

## Future Directions for Motor Rehabilitation

Imbalance of intra-limb coordination is one of the causes of motor impairment. If our hypothesis is true, muscle synergy (i.e., the balance of mechanical impedance) should be carefully taken into account when selecting methods of rehabilitation. The importance of impedance control has been discussed frequently, but care is required in its application because impedance control can result in either improved or worsened outcomes based on the way it is used. Muscle synergy may be an index for exploring the appropriate assistance application of impedance control. It would also provide an additional measure to clinical assessment such as the Fugl–Meyer assessment and others. Again, our hypothesis is that muscle synergies, the balance of mechanical impedance, represent a reference frame in the muscle space. This study tested our hypothesis to confirm the feasibility of the practical use of muscle synergy, such as in the assessment, diagnosis, and intervention planning for stroke rehabilitation.

The results of this study can be summarized as follows: (1) muscle synergy is an invariant balance of muscle mechanical impedance; (2) muscle synergies represent a reference frame in the muscle space; and (3) the common muscle synergies were found among different tasks (fast-spiral-tracing with the non-dominant hand and slow-circle-tracing with the dominant hand), different subjects from different generations (i.e., from subjects aged in their 20s to subjects aged in their 70s), and different levels of motor skill (beginner, experienced, and in a patient after rehabilitation). Further data collection and analysis from different situations will strengthen our hypothesis; it would be useful to discuss the relationship with other internal representations such as eye-centered, head-centered, and world-centered reference frames. Our future work includes the development of novel approaches for robotic therapy, particularly for the lower extremity. Robotic therapy, especially for lower extremity function, is currently in the early stage of development. The next generation of robot-aided neuro-rehabilitation requires correctly assessing the effect of interventions and collecting clinical evidence to develop an efficacious intervention. Stroke rehabilitation requires sensorimotor coordination. By combining the methods of *synergy assessment* and *robotic therapy*, we will develop a novel robotic intervention, test, and validate it in the framework of muscle synergies.

In conclusion, this study showed evidence that the muscle ­synergies play a central role in motor representation and internal model formation. Muscle synergies are not primitives but functional modules of mechanical impedance. The balance of muscle mechanical impedance is essential for motor control, learning, and recovery.

## Conflict of Interest Statement

The authors declare that the research was conducted in the absence of any commercial or financial relationships that could be construed as a potential conflict of interest.
